# Histone lysine demethylase 3B (KDM3B) regulates the propagation of autophagy via transcriptional activation of autophagy-related genes

**DOI:** 10.1371/journal.pone.0236403

**Published:** 2020-07-27

**Authors:** Hyeonsoo Jung, Sang-Beom Seo

**Affiliations:** Department of Life Science, College of Natural Sciences, Chung-Ang University, Seoul, Republic of Korea; Children's Cancer Institute Australia, AUSTRALIA

## Abstract

Autophagy, a self-degradative physiological process, is critical for homeostasis maintenance and energy source balancing in response to various stresses, including nutrient deprivation. It is a highly conserved catabolic process in eukaryotes and is indispensable for cell survival as it involves degradation of unessential or excessive components and their subsequent recycling as building blocks for the synthesis of necessary molecules. Although the dysregulation of autophagy has been reported to broadly contribute to various diseases, including cancers and neurodegenerative diseases, the molecular mechanisms underlying the epigenetic regulation of autophagy are poorly elucidated. Here, we report that the level of lysine demethylase 3B (KDM3B) increases in nutrient-deprived HCT116 cells, a colorectal carcinoma cell line, resulting in transcriptional activation of the autophagy-inducing genes. KDM3B was found to enhance the transcription by demethylating H3K9me2 on the promoter of these genes. Furthermore, we observed that the depletion of KDM3B inhibited the autophagic flux in HCT116 cells. Collectively, these data suggested the critical role of KDM3B in the regulation of autophagy-related genes via H3K9me2 demethylation and induction of autophagy in nutrient-starved HCT116 cells.

## Introduction

Autophagy is highly evolutionarily conserved and involved in several pathological pathways, including those related to cancers and neurodegenerative diseases [1]. Autophagy is a self-digestive catabolic process by which cellular materials, including proteins, organelles, and other cellular components, are degraded and recycled for energy balancing and generating molecular precursors. Macroautophagy has been widely studied and is considered to play critical roles in many diseases [2, 3]. Macroautophagy is induced as a response to various signals and stresses, most of which feed into the PI3K/mTORC1 pathway [4]. These signaling pathways regulate the core autophagy machinery involving over 30 regulators encoded by autophagy related genes (ATGs) [5]. The autophagy is initiated by the activation of ULK1 complex and PI3K Class III complex, which cause vesicle nucleation. The autophagosome membrane is expanded by the conjugation of the ATG5-ATG12 complex to ATG16 and LC3 is recruited to the membrane after the conjugation with lipid phosphatidylethanolamine (PE). ATG4B and ATG7 facilitate the conjugation of LC3B-I with PE leading to the formation of LC3B-II, which is commonly used as marker for autophagic flux [6]. The elements are degraded and recycled as precursors or fueled into the metabolic pathways in the autolysosome, the fused form of the autophagosome and the lysosome.

Autophagy has context-dependent roles, which either promote or inhibit the cell death in cancers [7, 8]. For example, autophagy can either enhance or reduce the apoptosis in the same tumor cell population under similar death stimuli [9]. Furthermore, most anticancer drugs as well as ionizing radiations are reported to increase autophagy in tumor cells; certain autophagy inhibitors, such as hydroxychloroquine (HCQ) and chloroquine (CQ), are already used in cancer treatment [8]. Several studies have warned about targeting autophagy for the treatment of cancers as this would reduce antitumor T cell responses [10, 11]. Therefore, the mechanisms underlying the autophagic flux must be investigated in depth to determine the appropriate trials required before the treatment can be used in the clinic; new substitutive therapeutic targets are also needed to avoid the side effects of autophagy regulation.

Although autophagy is a cytosolic event, several studies have revealed the importance of epigenetic regulation occurred in nucleus for proper autophagic flux [12–17]. Sustained starvation stress results in changes in the epigenetic networks, while acute response of autophagy is primarily observed in the cytoplasm. Various histone modifiers are reported to have crucial roles in autophagy. For example, CARM1, which catalyzes H3K17 methylation, activates autophagy and lysosomal genes under a state of glucose starvation [16]. G9a and EZH2 repress the expression of several autophagy-related genes by H3K9 methylation and H3K27 methylation, respectively, resulting in the suppression of autophagic flux [14, 17]. Regulation of H4K16 acetylation by acetyltransferase hMOF and deacetylase SIRT1 is associated with autophagy-related genes that determine cell survival [15]. H3K56 acetylation, H4K20 methylation, and H2BK120 monoubiquitination are also known to suppress the autophagy [12]. However, the mechanisms by which epigenetic pathways regulate autophagy are not well-elucidated.

KDM3B is a Jumonji C domain-containing protein that catalyzes demethylation of H3K9me1 and H3K9me2, resulting mostly in the activation of gene transcription [18]. Several studies have reported the physiological roles of KDM3B in different type of cells, including those from cancers and leukemia. KDM3B could regulate the expression of leukemogenic *lmo2* by reducing H3K9me2 on the promoter in HL-60, which are acute promyelocytic leukemia cells [18]. Furthermore, the demethylation activity of KDM3 family plays a crucial role in tumorigenic potential and survival of human colorectal cancer stem cells by the activation of the Wnt target gene [19]. A recent study has demonstrated the critical functions of KDM3B in the regulation of cell cycle and proliferation of HepG2 hepatocarcinoma cells [20]. However, the molecular mechanisms underlying the role of KDM3B in regulating cancers have not been well elucidated till date.

Valosin-containing protein (VCP)/p97 is an ATP-driven chaperone involved in various independent cellular processes [21]. VCP/p97 belongs to the hexameric ATPases associated with diverse cellular activities (AAA) family of proteins with two ATPase domains. Although VCP/p97 cannot directly catalyze the ubiquitination, it could structurally remodel the ubiquitinated proteins and edit the ubiquitin modification, which introduces an additional regulation and plasticity to ubiquitin-mediated systems [22, 23]. The regulation of ubiquitination by VCP/p97 governs cellular physiology and is relevant in various cancers and degenerative diseases [24].

In this study, we have identified the induction of KDM3B in the rapamycin-treated or nutrient-deprived colorectal carcinoma cell line, HCT116, caused by starvation media. We found that KDM3B is essential for proper autophagic propagation by regulating the transcription of autophagy-related genes via demethylation of H3K9me2 on the promoter of the genes in starved HCT116 cells. During autophagy, the interaction between KDM3B and VCP, which catalyzes the proteasomal degradation, was reduced, resulting in an increased level of KDM3B.

## Materials and methods

### Cell culture

HCT116 cells (KCBL10247, Korean cell line bank, Seoul, Korea) were grown in RPMI-1640 medium (Gibco, Grand Island, USA), and HEK293T cells (HC20005, Korean collection of type cultures, Jeongeup, Korea) were grown in Dulbecco’s modified Eagle’s medium (DMEM; Gibco, Grand Island, USA) containing 10% heat-inactivated fetal bovine serum (FBS; Gibco, Grand Island, USA) and 0.05% penicillin-streptomycin. HCT116 cells were acquired from laboratory of Dr. Kwang-Ho Lee, and HEK293T cells were purchased. Both the cell lines were maintained at 37°C in a 5% CO_2_ atmosphere. For induction of autophagy, HCT116 cells were treated with either starvation media, chloroquine (CQ; C6628, Sigma-Aldrich, Missouri, USA), and rapamycin (Glentham life science, Wiltshire, United kingdom) or DMSO (Duchefa Biochemie, Haarlem, Netherlands).

### Plasmid constructs

For the luciferase assay, the *ATG5* and *ATG7* promoter regions (−1510 to −7 and −1538 to -26, respectively) were amplified from genomic DNA using the primer pairs shown in S1 Table and inserted into the pGL3.0-basic vector (Promega, Madison, USA). pOTB7-VCP and pT7T3Pac-MAP1LC3B (Korea Unigene Clone) were subcloned into the pCMV-Flag vector and pEGFP-C1 vector, respectively, and Flag-VCP was subcloned into the plenti-puro vector (Addgene) using primer pairs mentioned in S1 Table. Short hairpin RNAs (shRNAs) against *KDM3B* and *VCP* were designed using siRNA sequence design software (Clontech). Double-stranded oligonucleotides for shRNA plasmid construction were produced using primers at the 5′ and 3′ ends (S1 Table). These oligonucleotides were inserted into the *Age*I/*EcoR*I site of the pLKO.1 TRC vector (Addgene).

### Antibodies

The antibodies used for the ChIP assays were directed against KDM3B (sc-101987, Santa Cruz Biotechnology, Dallas, USA), and H3K9me2 (07–441, Millipore, Massachusetts, USA). Antibodies against MMSET (sc-365627, Santa Cruz Biotechnology), LSD1 (sc-271720, Santa Cruz Biotechnology), p62 (sc-28359, Santa Cruz Biotechnology), β-actin (sc-47778, Santa Cruz Biotechnology), VCP (sc-20799, Santa Cruz Biotechnology), KDM2B (09–864, Millipore), DNMT1 (A300-041A, Bethyl Laboratories, Montgomery, USA), HDAC2 (ab12169, Abcam, Cambridge, United Kingdom), LC3B (2775S, Cell Signaling, Massachusetts, USA), Flag (F3165, Sigma-Aldrich, Missouri, USA), KDM3B (3314, Cell Signaling, Massachusetts, USA), ATG5 (12994S, Cell Signaling, Massachusetts, USA), mTOR (ABP51867, Abbkine, Wuhan, China), and mTOR S2448ph (ABP50363, Abbkine, Wuhan, China) were used in the immunoblot assays.

### Reverse transcription polymerase chain reaction (PCR) and quantitative real-time PCR (qPCR)

Total RNA was isolated from cells using Tri-RNA Reagent (Favorgen, Ping-Tung, Taiwan,). After its synthesis, the cDNA was quantified and subjected to mRNA expression analysis. The PCR primers used are given in S1 Table. Dissociation curves were examined after each PCR run to ensure the amplification of a single product of appropriate length. The mean threshold cycle (C_T_) and standard error values were calculated from individual C_T_ values obtained from three independent reactions per stage. The mean normalized C_T_ value was estimated as ΔC_T_ by subtracting the mean C_T_ of β-actin. The ΔΔC_T_ value was calculated as the difference between the control ΔC_T_ and the value obtained for each sample. The n-fold change in gene expression relative to the expression of a control, was calculated as 2^-ΔΔCT^.

### Lentivirus transduction

To produce the virus particles, HEK293T cells were co-transfected with plasmids encoding VSV-G, NL-BH, and plenti-Flag-VCP or shRNAs against *KDM3B* and *VCP*. Two days after the transfection, supernatants containing the virus were collected and used to infect HCT116 cells in the presence of polybrene (8 μg/mL).

### Luciferase assay

For the luciferase assay, stable sh*KDM3B* HCT116 cells were seeded in 48-well plates and co-transfected with the indicated expression plasmid and pGL3.0-*ATG5* or pGL3.0-*ATG7* reporter plasmid using polyethyleneimine. After 48 hours, the cells were harvested and subjected to the luciferase assay (Promega). β-galactosidase activity levels were used to normalize the reporter luciferase activity. Data are expressed as the mean of four replicates in a single assay. All results shown are representative of at least three independent experiments.

### ChIP-qPCR assay

Cells were harvested and subsequently cross-linked with 1% formaldehyde. Briefly, 1% formaldehyde was added to the medium for 10 min at room temperature, followed by the addition of 125 mM glycine for 5 min at room temperature. HL-60 cells were centrifuged and the resulting pellets were washed once with 1× phosphate-buffered saline (PBS). The cell pellets were resuspended in sodium dodecyl sulfate (SDS) lysis buffer (1% SDS, 10 mM EDTA, 50 mM Tris-HCl [pH 8.1]). The cells were sonicated, and the lysates were subjected to IP using the indicated antibodies. The immunoprecipitants were eluted and reverse cross-linked. Subsequently, the DNA fragments were purified and PCR amplified for quantification using the designated PCR primer pair (S1 Table). The thermal cycling conditions were as follows: 3 min at 95 °C followed by 45 cycles of 95 °C for 10 s, 58 °C for 10 s, and 72 °C for 30 s (Bio-Rad). The mean threshold cycle (C_T_) and standard error values were calculated from individual C_T_ values from duplicate reactions at each stage.

### Immunoprecipitation

IP was performed to investigate the relationship between KDM3B and VCP in untreated or rapamycin-treated HCT116 cells. Stably Flag-VCP overexpressing HCT116 cells were treated with 2 μM of rapamycin for 8 hours and lysed in a lysis buffer (20 mM Tris-HCl [pH 7.5], 150 mM NaCl, 1 mM EDTA, 1 mM EGTA, 1% Triton X-100, 1× protease inhibitor cocktail, and 1 mM PMSF) at 4 °C after 2 hours of incubation. Proteins were immunoprecipitated with anti-KDM3B antibodies or Flag antibodies overnight at 4 °C. Subsequently, protein A/G agarose beads (GenDEPOT, Katy, USA) were added to it and the samples were incubated for 4 hours with rotation at 4 °C. Bound proteins were analyzed via western blotting with anti-KDM3B and anti-Flag antibodies.

### Detection of GFP-LC3B puncta

Stable sh*KDM3B* cells cultured on coverslips were treated with 2 μM rapamycin for 8 hours. After washing with PBS, cells were fixed in 4% paraformaldehyde (Electron Microscopy Science, Hatfield, USA) for 5 min. 4’,6-diamidino-2-phenylindole (DAPI) staining was performed to visualize nuclear DNA. The coverslips were mounted onto glass slides and visualized using a Nikon ECLIPSE Ti2 inverted microscope system. The number of GFP-LC3B puncta were counted using Image J.

### MTT assay

shNC and sh*KDM3B* HCT116 cells were seeded in 48-well plates with 8 × 10^4^ per each well. After 24, 48, and 72 h, MTT ((3-(4,5-dimethylthiazol-2-yl)-2,5-diphenyltetrazolium bromide) was added to the cells (final concentration 0.5 mg/ml), after which they were incubated further for 4 h at 37 °C. The medium was then removed by aspiration, and DMSO was added (200 μl/well), and The OD was determined using a microplate spectrophotometer (BioTek, Winooski, USA) at the wavelength of 585 nm.

### Statistical analysis

Data are expressed as the mean ± standard deviation for the ChIP assay and mean ± standard error of the mean for the gene expression and luciferase assays, based on three or more independent experiments. Statistical significance (*P* < 0.05) was calculated using Microsoft Excel and GraphPad Prism (version 8.3; GraphPad software, San Diego, USA). Differences between the groups were evaluated by Student’s t-test or Bonferroni test, as appropriate.

## Results

### The level of KDM3B increases during autophagic flux without changes in the global histone modifications

**Fig 1:**
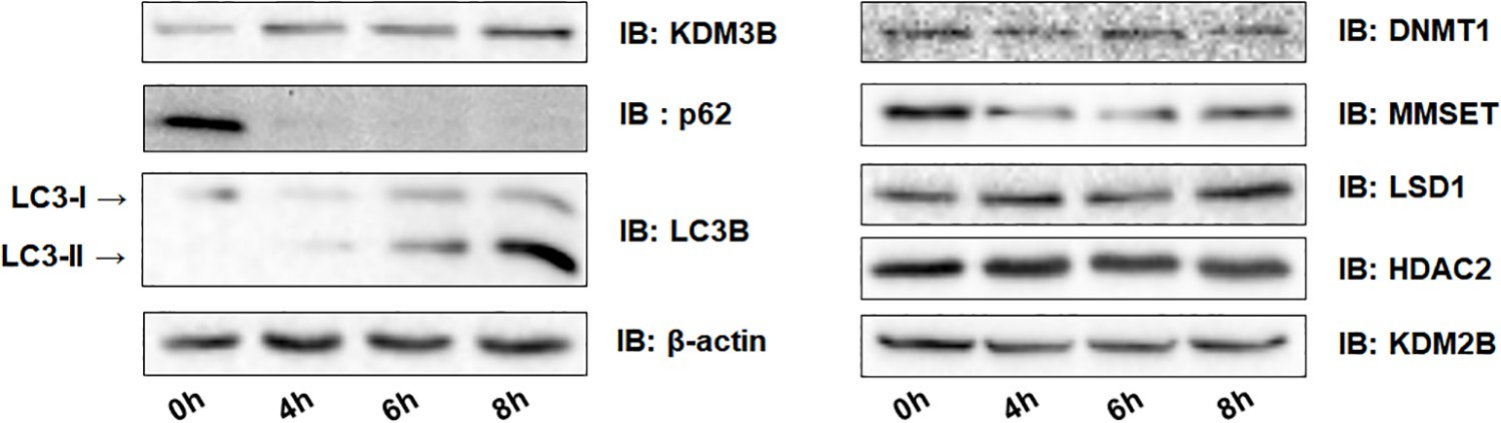
The changes of the expression of epigenetic enzymes in nutrient-deprived HCT116 cells. HCT116 cells were starved using the starvation medium. After 4, 6, and 8 hours, cells were harvested. Extracts were eluted, resolved by SDS-PAGE, and immunoblotted with each specified antibody.

To figure out the epigenetic mechanisms underlying autophagic flux, we compared the protein expression levels of several epigenetic modifiers in amino acid starved HCT116 cells using starvation medium (Fig 1) [25]. We tested DNA methyltransferase DNMT1, H3K36 methyltransferase MMSET, H3K4 and H3K9 demethylase LSD1, histone deacetylase HDAC2, H3K36 and H3K79 demethylase KDM2B, and H3K9 demethylase KDM3B [18, 20, 26–30]. Markers of autophagy, p62/SQSTM1 degradation, and conversion of LC3B-I to LC3B-II were also detected [6, 31]. Although epigenetic enzymes either decreased or showed no significant changes, the level of KDM3B was found to have increased after 4 hours in starvation medium. The induction of KDM3B in starved HCT116 cells suggested its potential role in autophagic flux.

### KDM3B regulates the transcription of autophagy-related genes

**Fig 2:**
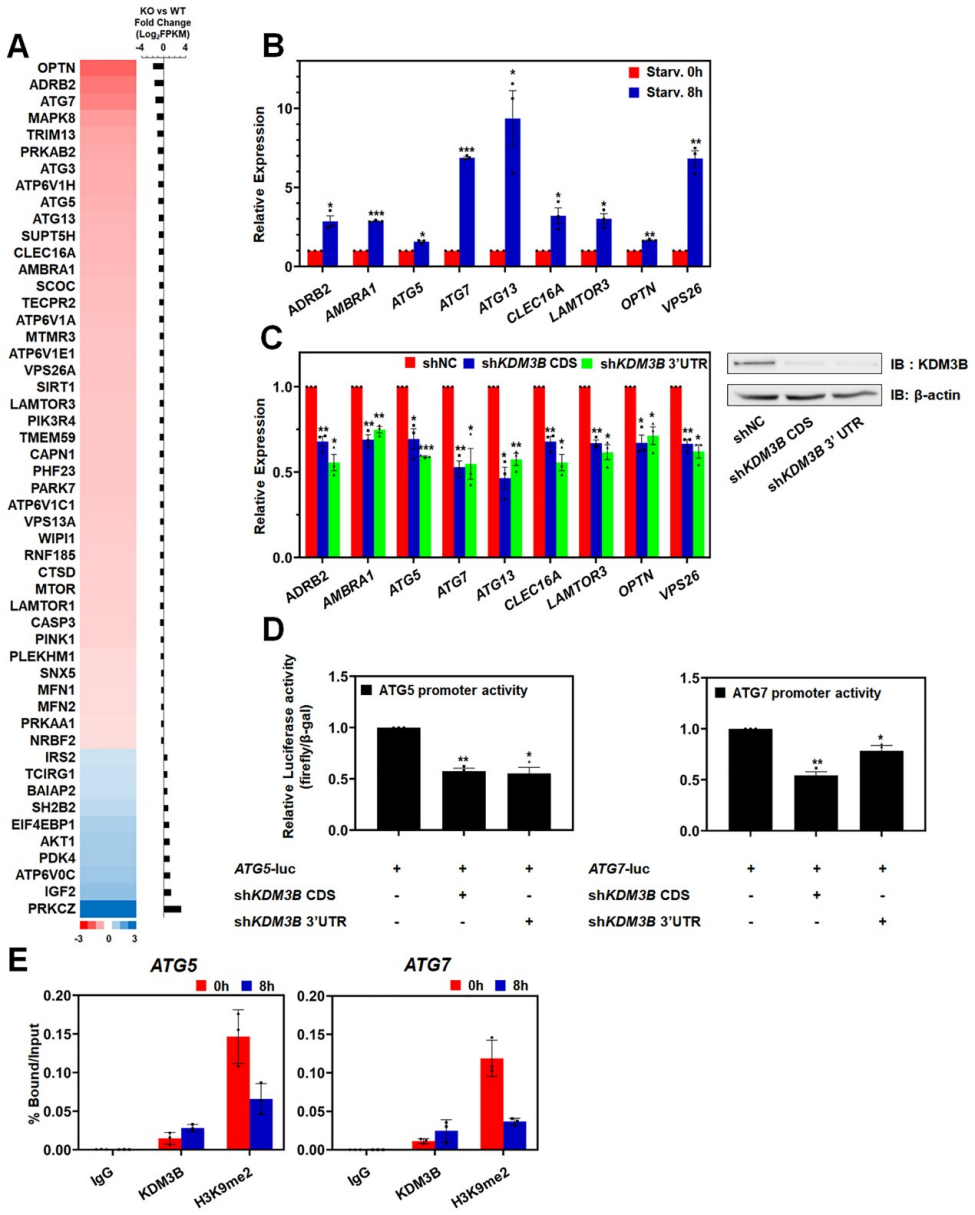
KDM3B regulates the transcription of autophagy-related genes. (A) The differential expression of autophagy-related genes in KDM3B wildtype and KO HepG2 cells was visualized and analyzed. (B-C) The changes in target gene expression were measured by qPCR. (B) HCT116 cells were incubated in starvation medium for 8 hours. (C) Stable sh*KDM3B* CDS and sh*KDM3B* 3′UTR HCT116 cells were harvested and RNA of the cells was isolated. Knockdown of KDM3B was confirmed by immunoblotting. (D) Stable sh*KDM3B* HCT116 cells were co-transfected with pGL3.0-*ATG5* or pGL3.0-*ATG7* promoters. Luciferase activities were measured 48 hours after transfection and normalized to that of β-galactosidase. (B-D) All results represent the data from at least three independent experiments (± SEMs). * *P* < 0.05, ** *P* < 0.01, *** *P* < 0.001. (E) The occupancies of KDM3B and H3K9me2 at the promoters of autophagy-related genes in nutrient starved-HCT116 cells were analyzed. The data were normalized by input. The results are shown as mean ± SD (n = 3).

Generally, KDM3B acts as a transcriptional activator via demethylating H3K9. To investigate the functions of KDM3B in autophagic flux, we analyzed the RNA-seq database in KDM3B Knockout (KO) HepG2 cells (Fig 2A) [20]. Several autophagy-inducing genes were repressed in KDM3B KO HepG2 cells, while autophagy-inhibiting genes were induced. To validate this data in HCT116 cells, we constructed stable *KDM3B*-depleted HCT116 cells using shRNAs. First, we confirmed the induction of autophagy-inducing genes in starved HCT116 cells (Fig 2B). ATG5, ATG7, and ATG13 are the critical regulators of the autophagy pathways [5]. Moreover, adrenoceptor β2 (ADRB2), activating molecules in Beclin 1-regulated autophagy protein 1 (AMBRA1), C-type lectin domain containing 16A (CLEC16A), late endosomal/lysosomal adaptor, MAPK and mTOR activator 3 (LAMTOR3), optineurin (OPTN), and vacuolar protein sorting associated protein 26A (VPS26A) were also reported to have critical roles in autophagy [32–37]. Interestingly, the target genes were attenuated in stable sh*KDM3B* HCT116 cells (Fig 2C). Consistent with RNA-seq data, KDM3B was able to regulate the gene expression in HCT116 cells. We also confirmed the expression level of ATG5 in stable sh*KDM3B* HCT116 cells by immunoblot. As well known, ATG5 increased during autophagy induced by starvation (S1A Fig). In sh*KDM3B* HCT116 cells, the expression of ATG5 was reduced (S1B Fig). However, starvation resulted in the moderate induction of ATG5, which suggested the importance of KDM3B in ATG5 transcription and the possibility of the other compensative pathways which could modulate the ATG5 expression. These data suggested that KDM3B could modulate the autophagy by activating the autophagy-inducing genes epigenetically in starved HCT116 cells.

Next, we performed luciferase assay to identify whether KDM3B regulated the expression of target genes at the transcriptional level. We transiently overexpressed the luciferase vector containing the promoter of *ATG5* and *ATG7* in stable sh*KDM3B* HCT116 (Fig 2D). The data showed that KDM3B could regulate the transcription of the genes. Because several studies showed that diverse histone modifications including H3K9me2 were not changed during autophagic flux, we hypothesized that the KDM3B might regulates the H3K9me2 levels on the promoters of specific target genes. The ChIP-qPCR data using H3K9me2 and KDM3B antibodies in starved HCT116 cells indicated that KDM3B is recruited to and decreases H3K9me2 on the promoters of the target genes (Fig 2E). Taken together, KDM3B could bind to the promoters of the autophagy-inducing genes and activate the transcription of these genes by demethylation of H3K9 on the promoter region during starvation-induced autophagy in HCT116 cells.

### Rapamycin induces KDM3B in amino acid starved HCT116 cells

**Fig 3:**
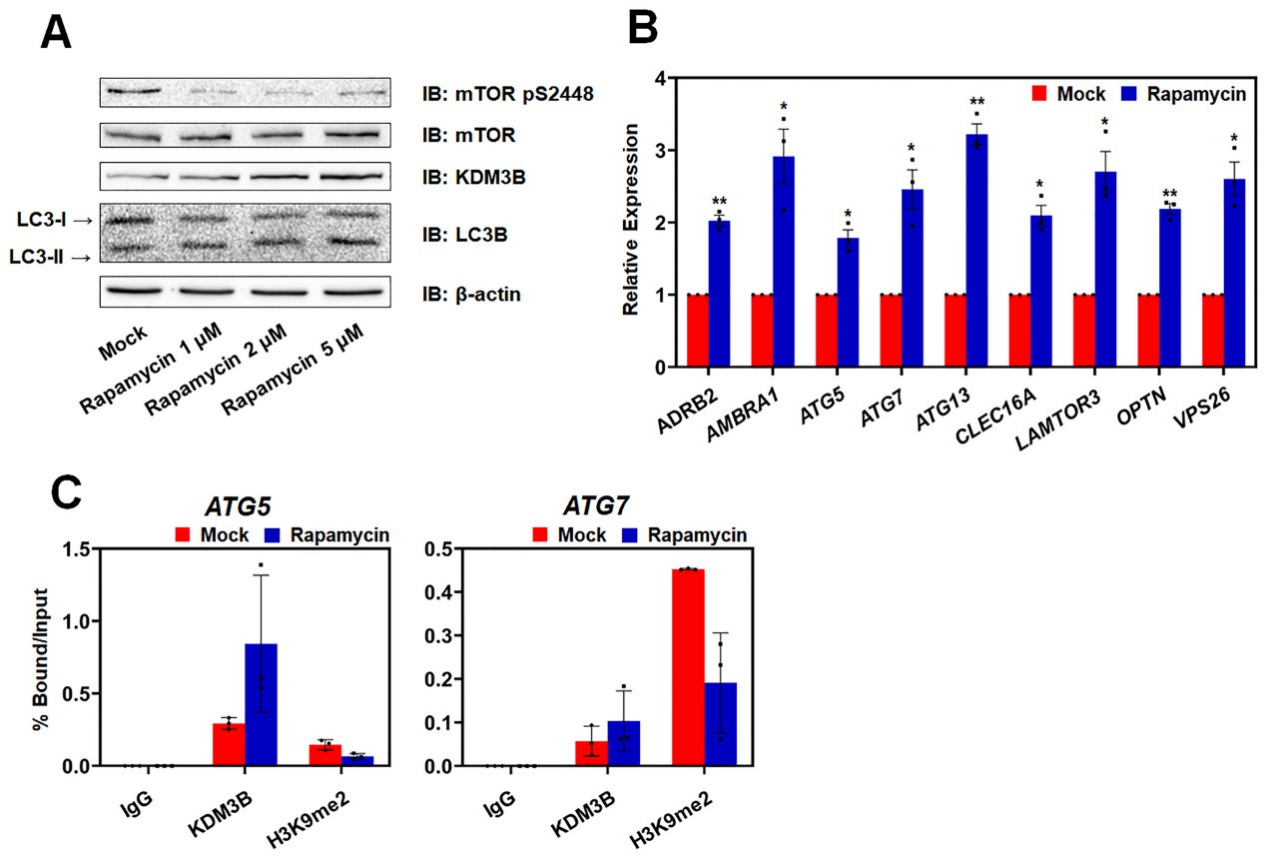
Rapamycin increased KDM3B recruitment to the promoters of autophagy-related target genes. (A) HCT116 cells were treated with 1, 2, or 5 μM of rapamycin or DMSO for 8 hours. Cell extracts were eluted and immunoblotted using KDM3B antibody and LC3B. Antibodies against mTOR and mTOR pS2448 were used to confirm the activity of rapamycin. (B) mRNA levels of autophagy-related genes in HCT116 cells treated with 2 μM of rapamycin for 8 hours were confirmed by qPCR. All results represent the data obtained from at least three independent experiments (± SEMs). * *P* < 0.05, ** *P* < 0.01. (C) ChIP analyses of the promoters of the target genes in HCT116 cells treated with 2 μM of rapamycin for 8 hours were examined using antibodies against KDM3B and H3K9me2 and rabbit IgG by qPCR. The results are shown as mean ± SD (n = 3).

Various autophagy inducers can lead to different effects [16]. We tested another autophagy inducer, rapamycin, which is one of the autophagy inducers that broadly act via inhibiting mTOR activity [38]. Because mTOR inhibition or rapamycin is considered as the inducer of autophagy initiation, we treated the cells with rapamycin and detected the level of KDM3B in HCT116 cells to identify whether KDM3B acts downstream of mTOR inhibition signaling in the autophagic flux (Fig 3A). Rapamycin treatment reduced the phosphorylation of mTOR at S2448, which is consistent with the previous studies, and induced a KDM3B level similar to that observed after starvation. Furthermore, rapamycin could enhance the transcription of the genes that were activated by starvation and KDM3B under HCT116 starvation conditions (Fig 3B). When the cells were treated with rapamycin, KDM3B was recruited to and removed H3K9me2 on the promoter of the autophagy-related genes (Fig 3C). Taken together, rapamycin treatment also enhanced the KDM3B expression and its recruitment to the promoter of the target genes resulting in the transcriptional activation of the genes by demethylation of H3K9me2.

### KDM3B is crucial for the proper progression of autophagy

**Fig 4:**
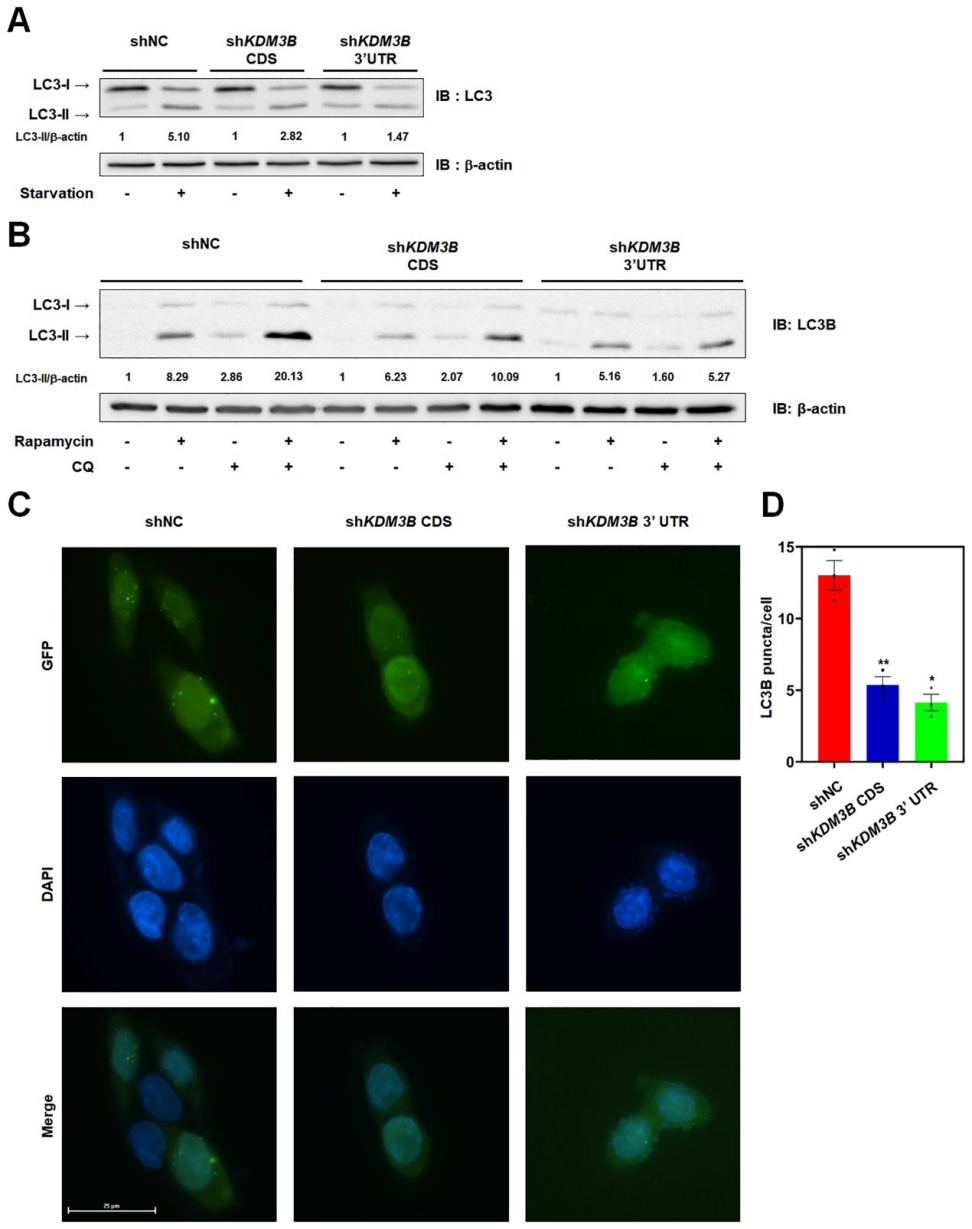
KDM3B is a prerequisite for the autophagic flux. (A-B) The proteins from the stable sh*KDM3B* HCT116 cells were extracted, resolved by SDS-PAGE, and immunoblotted with an anti-LC3B antibody to measure the ratio of the conversion of LC3B-I to LC3B-II. The ratio of LC3B conversion was measured by Image J. (A) Cells were incubated in starvation medium for 8 hours. (B) *KDM3B* depleted HCT116 cells were treated with 2 μM rapamycin for 8 hours. CQ was also added to the cells for 4 hours. (C) The formations of the GFP-LC3B puncta were measured by fluorescent microscopy in GFP-LC3B overexpressed HCT116 cells treated with 2 μM rapamycin for 8 hours. (D) The GFP-LC3B puncta per cells were measured by Image J. The numbers of GFP-LC3B puncta were counted at least 5 cells, and this data represent the data obtained from at least three independent experiments (± SEMs). * *P* < 0.05, ** *P* < 0.01.

We have investigated the induction of KDM3B and activity of it to activate transcription of autophagy-related genes in nutrient-deprived or rapamycin treated HCT116 cells. To confirm whether KDM3B was a prerequisite for autophagy, we detected the autophagic flux in stable sh*KDM3B* HCT116 cells. As expected, *KDM3B* depleted HCT116 cells showed lower LC3-II conversion ratio when starved or treated with rapamycin and CQ (Fig 4A and 4B). Autophagy inhibitor, CQ, which impairs autophagosome fusion with lysosome and disrupts lysosomal degradation, causes the accumulation of LC3B-II [39]. Treatment of Rapamycin with CQ confirmed the disrupted autophagic flux in sh*KDM3B* HCT116 cells (Fig 4B). We could also detect the defects in LC3B conversion in GFP-LC3B overexpressed sh*KDM3B* HCT116 cells using fluorescence microscopy (Fig 4C and 4D). *KDM3B* depletion suppressed the formation of LC3B puncta indicating that the knockdown of *KDM3B* inhibits the autophagic flux.

### Autophagy inhibited the degradation of KDM3B by VCP/p97

**Fig 5:**
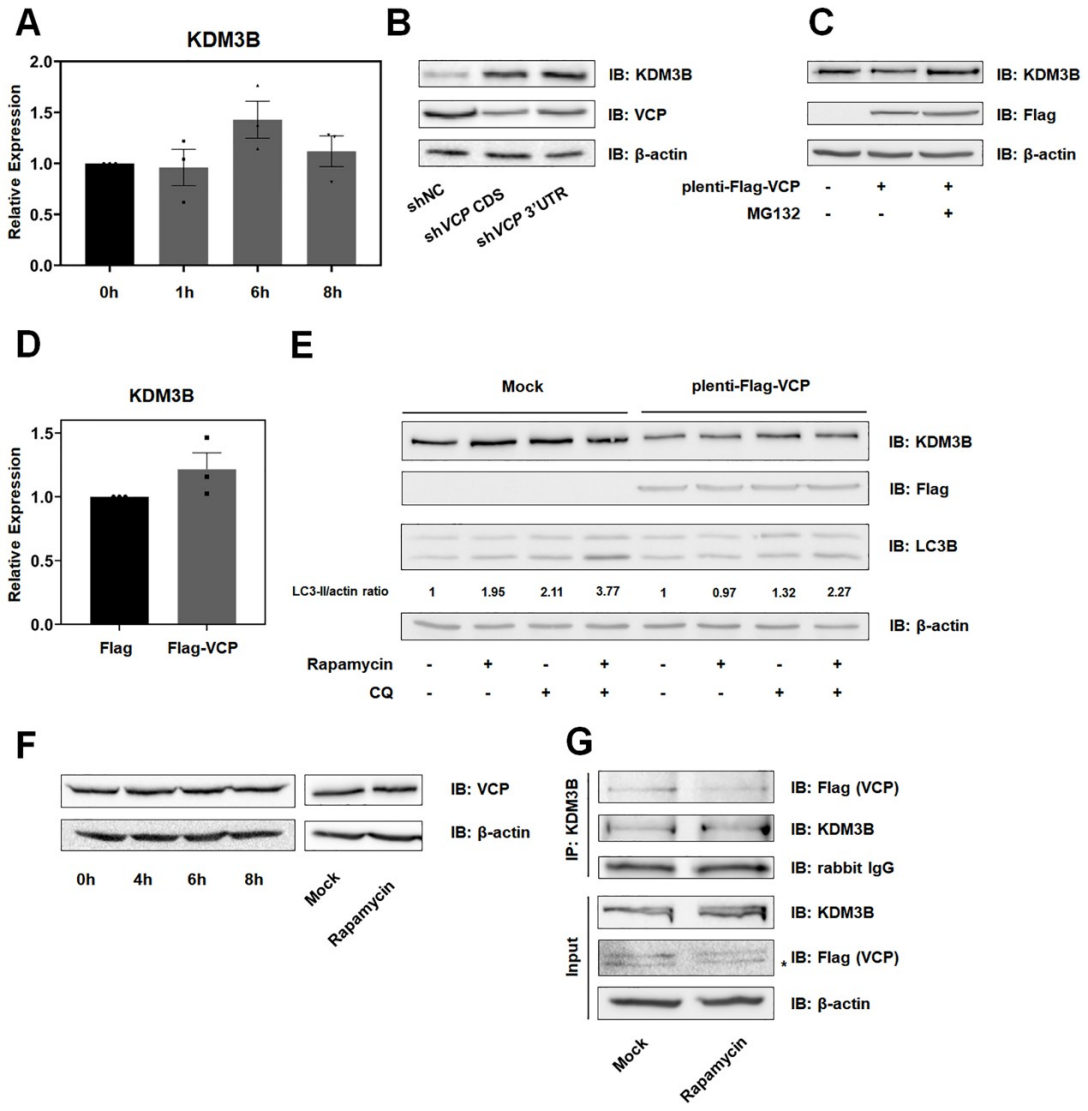
Autophagy inhibits the proteasomal degradation of KDM3B induced by VCP/p97 in HCT116 cells. (A) mRNA levels of *KDM3B* in HCT116 cells incubated in starvation medium for 4, 6, and 8 hours were confirmed by qPCR. (B) Stable *VCP*-depleted HCT116 cells were harvested (C) Mock and plenti-Flag-VCP HCT116 cells were treated with 20 μM of MG132 or DMSO for 4 hours. (B-C) Cell extracts were eluted and immunoblotted using antibodies against KDM3B and VCP or Flag. (D) The *KDM3B* transcript level was measured by qPCR in Flag-VCP-overexpressing HCT116 cells. (E) Mock and plenti-Flag-VCP overexpressing HCT116 cells were treated with rapamycin for 8 hours and/or CQ for 4 hours. The cell extracts were resolved by SDS-PAGE and immunoblotted with anti-KDM3B, anti-LC3B, and anti-Flag antibodies. The ratio of LC3B conversion was measured by Image J. (F) The protein level of VCP in HCT116 cells incubated with starvation media or treated with 2 μM of rapamycin for 8 hours, was measured by immunoblotting. (G) plenti-Flag-VCP-overexpressing HCT116 cells were treated with 2 μM of rapamycin for 8 hours and the cell extracts were immunoprecipitated with anti-KDM3B antibodies. The changes in the differential interaction were detected by immunoblotting with anti-KDM3B and anti-Flag antibodies. * indicates non-specific bands.

We found that the level of KDM3B increased during autophagy caused by nutrient-starvation or rapamycin treatment, which subsequently activated the transcription of autophagy-related genes and autophagic flux. To analyze how KDM3B was induced by starvation, we tested the mRNA level of *KDM3B* during autophagy (Fig 5A). However, we observed no significant changes in the level of *KDM3B* transcription in the nutrient-deprived HCT116 cells. Therefore, we hypothesized that KDM3B could be regulated by proteasomal degradation and looked for a putative partner that could catalyze the degradation of KDM3B. Through a literature review and a search on the BioGRID database (https://thebiogrid.org), we selected the ATPase VCP/p97 as a putative interacting partner. VCP can interact with KDM3B and has various roles, including its control over the ubiquitin-dependent protein degradation [21–24]. First, we confirmed the interactions between VCP and KDM3B in Flag-VCP overexpressing HCT116 cells (S2A and S2B Fig). To investigate the role of VCP in KDM3B regulation, we constructed stable sh*VCP* HCT116 cells using shRNAs targeting VCP and found that KDM3B was induced with VCP depletion (Fig 5B). Next, we lentivirally overexpressed Flag-tagged VCP in HCT116 cells to confirm whether VCP led to the degradation of KDM3B (Fig 5C). As expected, the overexpression of VCP reduced the level of KDM3B in HCT116 cells; however, the mRNA level of *KDM3B* did not change (Fig 5C and 5D). Furthermore, treatment with MG132, an inhibitor of proteasomes, recovered the KDM3B level, suggesting that the VCP can catalyze the proteasomal degradation of KDM3B (Fig 5C).

Since the role of VCP in autophagy has not been well elucidated, we sought to investigate whether VCP was critical in starved HCT116 cells by detecting LC3B conversion in stable Flag-VCP-overexpressing HCT116 cells (Fig 5E). Flag-VCP also impeded the autophagic flux through degradation of KDM3B in rapamycin and CQ treated HCT116 cells. These data indicated that VCP was involved in autophagic flux via the regulation of KDM3B in HCT116 cells.

To investigate the underlying mechanism of VCP in the regulation of KDM3B during autophagy, we tested the change in the expression level of *VCP* in nutrient-starved and 2 μM of rapamycin treated HCT116 cells (Fig 5F). The mRNA level of *VCP* was not changed during autophagy in HCT116 cells (S2C Fig). Thus, we hypothesized that the functions, and not the expression, might be changed during autophagy. As expected, interaction between KDM3B and VCP was reduced with rapamycin treatment (Fig 5G). These data indicated that VCP induced the proteasomal degradation of KDM3B in HCT116 cells; however, VCP could not attenuate the level of KDM3B, as the interaction between VCP and KDM3B was inhibited during autophagy.

Taken together, VCP catalyzes proteasomal degradation of KDM3B in HCT116 cells. Nutrient starvation or rapamycin treatment can lead to a decrease in the interaction between VCP and KDM3B, which can then induce the maintenance of KDM3B. Increase in the KDM3B level is a prerequisite for the appropriate propagation of autophagy through the activation of the transcription of autophagy-related genes.

## Discussion

In this study, we demonstrated the epigenetic regulation of KDM3B for the appropriate propagation of autophagy caused by starvation and mTOR inhibition. We observed that VCP could regulate the ubiquitination systems and induce proteasomal degradation of KDM3B in HCT116 cells, which represses autophagic flux under the normal state. When cells were starved or treated with rapamycin, the interaction between KDM3B and VCP diminished resulting in an increase in KDM3B levels. KDM3B was able to enhance the transcription of autophagy-related genes that activate the autophagy progression in nutrient-deprived or mTOR-inhibited HCT116 cells. Our data suggests that KDM3B is a critical regulator of autophagy in cancer.

Due to the side-effects of direct targeting of critical cellular processes for cancer therapies, the combination of chemotherapies or ionizing radiation with epigenetic regulation has been studied recently [40–42]. All-*trans* retinoic acid, which induces the differentiation of acute myeloid leukemia and is used in leukemia treatment, has been studied in combination with several epigenetic regulators, including inhibitors of HDAC and LSD1 [42, 43]. Although autophagy enhances the survival of cancer cells, the effects of targeting the autophagy in cancer therapy are not well understood. Epigenetic regulation could be a new putative therapeutic mechanism to avoid the risks of this strategy.

We observed that the depletion of KDM3B reduced the proliferation of HepG2 cells [20]. We also confirmed similar effects in HCT116 cells (S3 Fig). Furthermore, inhibition of autophagy in HCT116 by *ATG5* knockdown attenuated tumor growth *in vivo*. These data indicate the oncogenic roles of KDM3B in cancer, and maintenance of autophagy by KDM3B could function to promote the oncogenesis. Although CQ and HCQ, which are known to inhibit autophagy, particularly the associated lysosomal function, are used in cancer therapy, autophagy inhibition could cause the reduction of antitumor T cell responses and interfere with a robust antitumor immune response [8, 44]. Our study suggests KDM3B as a new therapeutic candidate, which enhances survival of cells and autophagy in cells. However, the circumstantial relationship between cell proliferation and autophagy in HCT116 cells regulated by KDM3B should be investigated further.

## Supporting information

S1 Fig ATG5, one of the important regulators of autophagy, was reduced in sh*KDM3B* HCT116 cells. (A) ATG5 was induced by starvation in HCT116 cells. (B) Stable sh*KDM3B* and control HCT116 cells were incubated with starvation media for 8 hours. The proteins from the cells were extracted, resolved by SDS-PAGE, and immunoblotted with an anti-ATG5 antibody.
(TIF)

S2 Fig The expressions of VCP mRNA were not changed by starvation. (A-B) plenti-Flag-VCP-overexpressing HCT116 cells were harvested and the cell extracts were immunoprecipitated with anti-KDM3B and anti-Flag antibodies. The interactions between KDM3B and VCP were detected by immunoblots. * indicates non-specific bands. (C) HCT116 cells were treated with starvation media for 4, 6, and 8 hours. The mRNA levels of VCP in HCT116 cells were confirmed by qPCR. The results represent at least three independent experiments (± SEMs).
(TIF)

S3 Fig KDM3B is critical for maintaining of cell proliferation in HCT116 cells. Cell proliferation was accessed through MTT assays in stable *KDM3B* knockdown HCT116 cells. The result is expressed as means ± SEM. (n = 3). * *P* < 0.05.
(TIF)

S4 Fig Original blot images in this study. (PDF)

S1 Table Primers used in this study. (XLSX)

S2 Table Minimal data set in this study. (XLSX)
