# Supplementary figures and images for: Histone lysine demethylase 3B (KDM3B) regulates the propagation of autophagy via transcriptional activation of autophagy-related genes
Source: PLoS One. 2020 Jul 27;15(7):e0236403. doi: 10.1371/journal.pone.0236403 (PMC7384621; doi:10.1371/journal.pone.0236403)

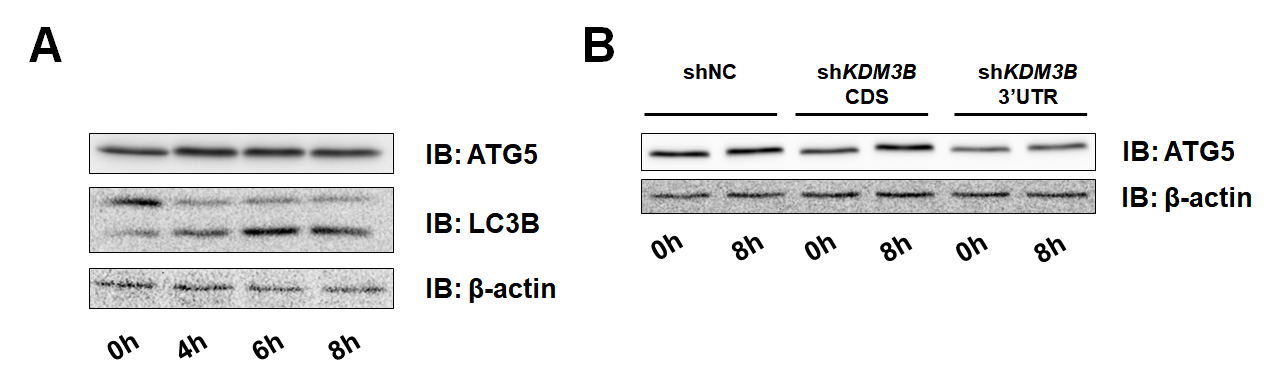

Supplement: S1 Fig — (A) ATG5 was induced by starvation in HCT116 cells. (B) Stable shKDM3B and control HCT116 cells were incubated with starvation media for 8 hours. The proteins from the cells were extracted, resolved by SDS-PAGE, and immunoblotted with an anti-ATG5 antibody. (TIF) [file pone.0236403.s001.tif]

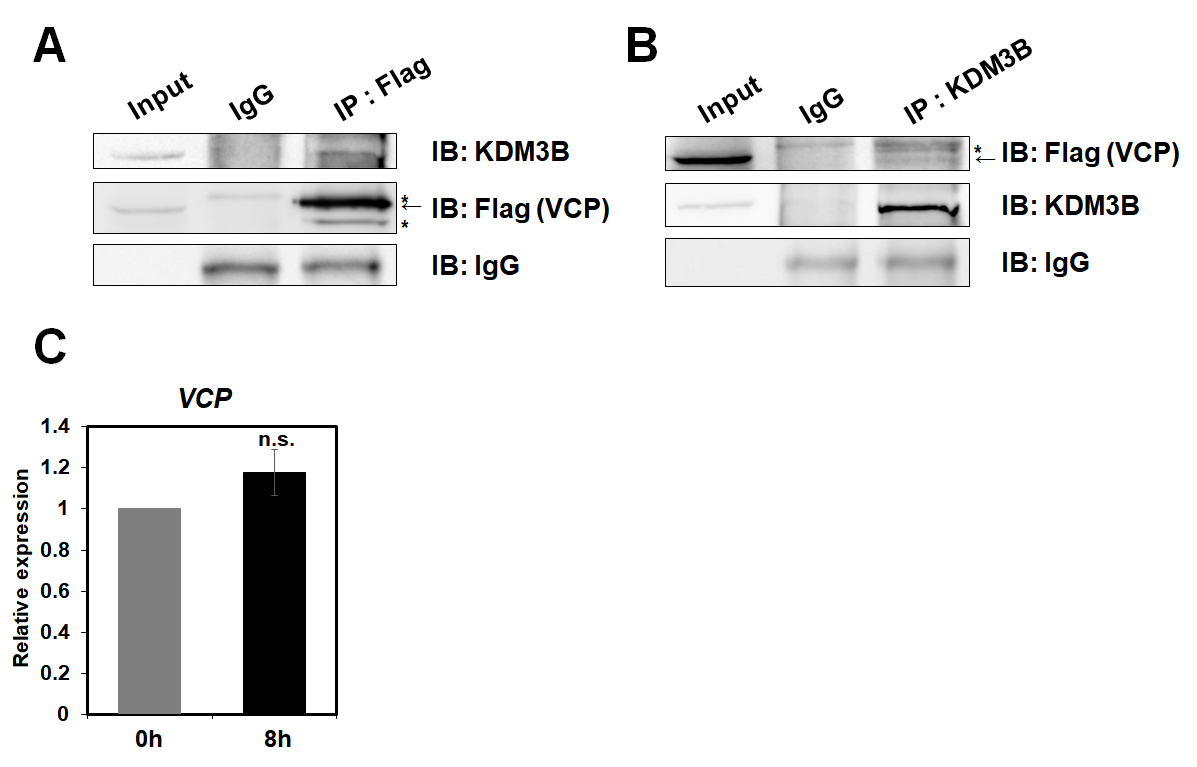

Supplement: S2 Fig — (A-B) plenti-Flag-VCP-overexpressing HCT116 cells were harvested and the cell extracts were immunoprecipitated with anti-KDM3B and anti-Flag antibodies. The interactions between KDM3B and VCP were detected by immunoblots. * indicates non-specific bands. (C) HCT116 cells were treated with starvation media for 4, 6, and 8 hours. The mRNA levels of VCP in HCT116 cells were confirmed by qPCR. The results represent at least three independent experiments (± SEMs). (TIF) [file pone.0236403.s002.tif]

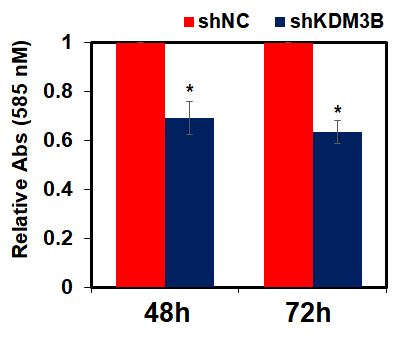

Supplement: S3 Fig — Cell proliferation was accessed through MTT assays in stable KDM3B knockdown HCT116 cells. The result is expressed as means ± SEM. (n = 3). * P < 0.05. (TIF) [file pone.0236403.s003.tif]
