# Supplementary material for: Histone lysine demethylase 3B (KDM3B) regulates the propagation of autophagy via transcriptional activation of autophagy-related genes
Source: PLoS One. 2020 Jul 27;15(7):e0236403. doi: 10.1371/journal.pone.0236403 (PMC7384621; doi:10.1371/journal.pone.0236403)

# Figure 1

## A The expression changes of proteins during starvation – Western blot analysis

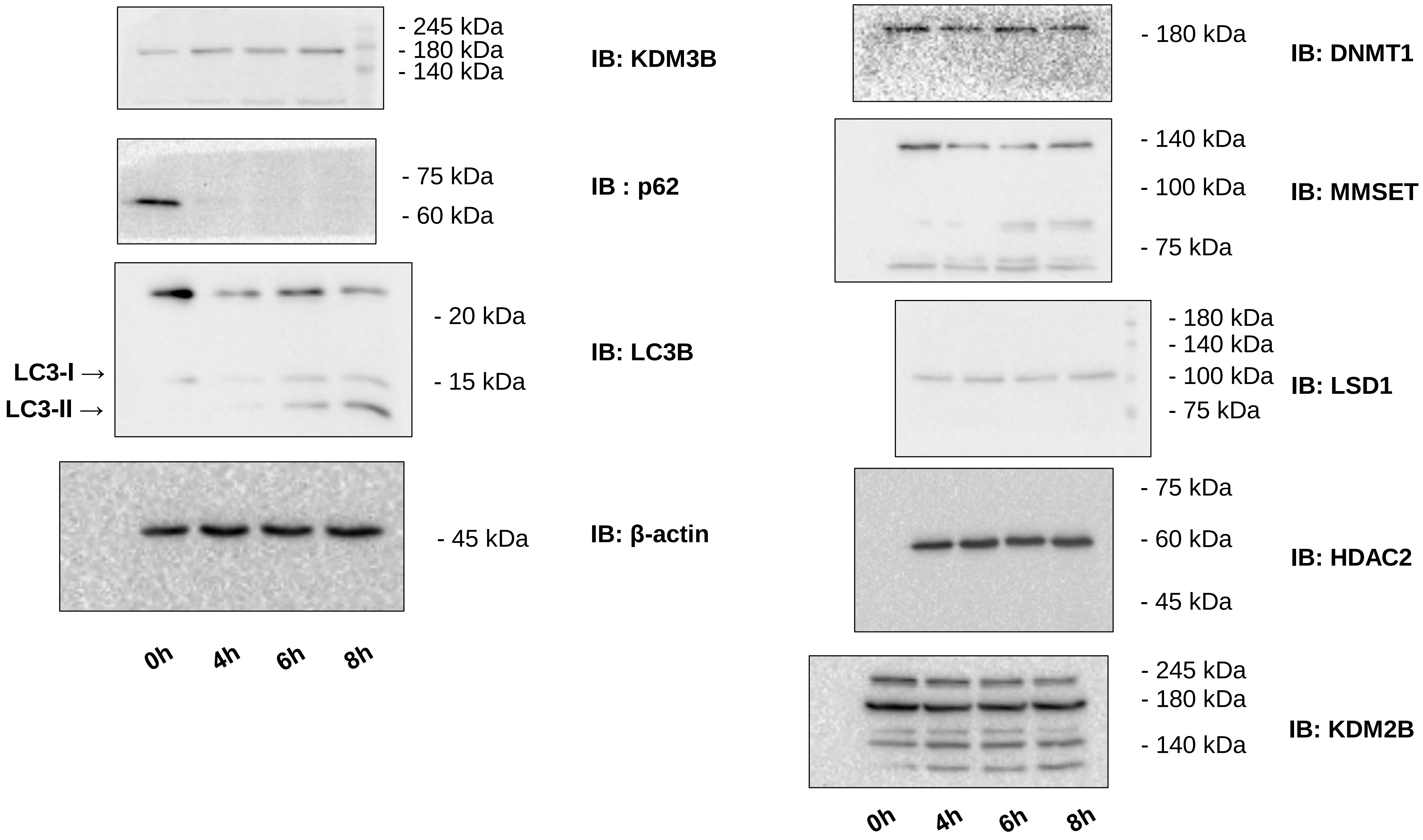

# Figure 2

**C** **Knockdown of KDM3B – Western blot analysis**

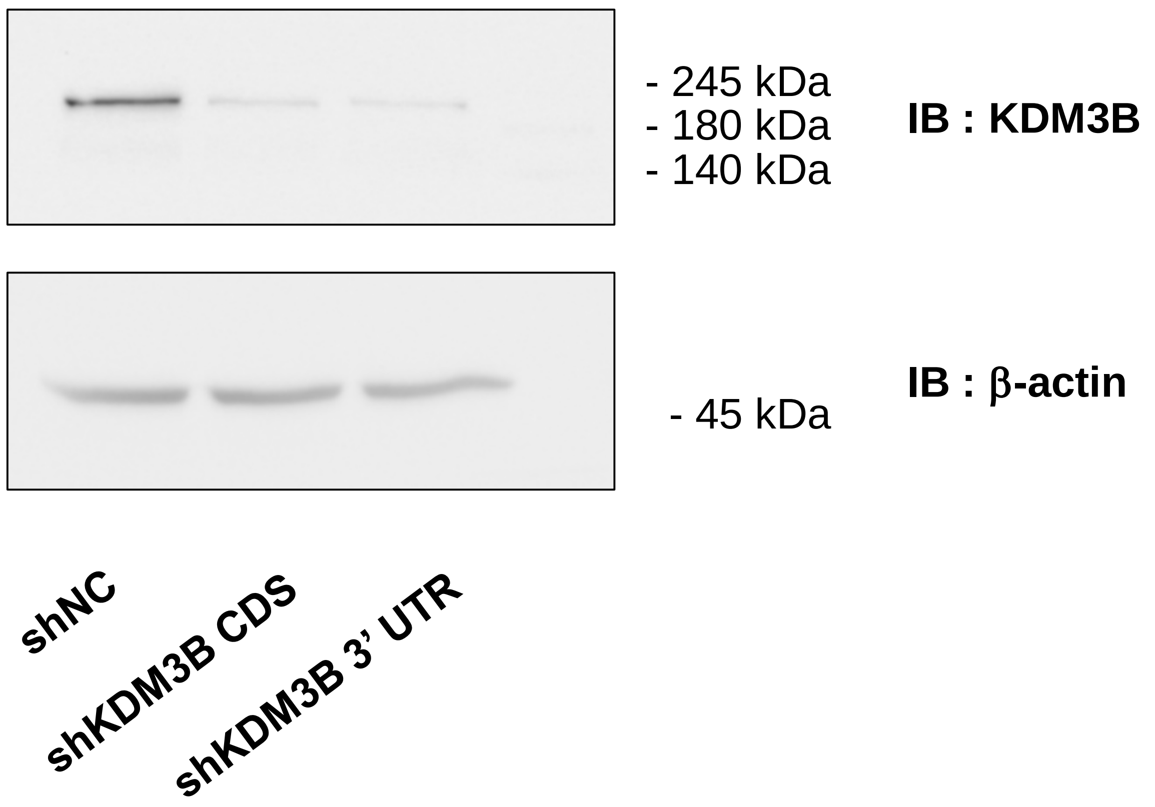

# Figure 3

## A The induction of KDM3B by rapamycin treatment – Western blot analysis

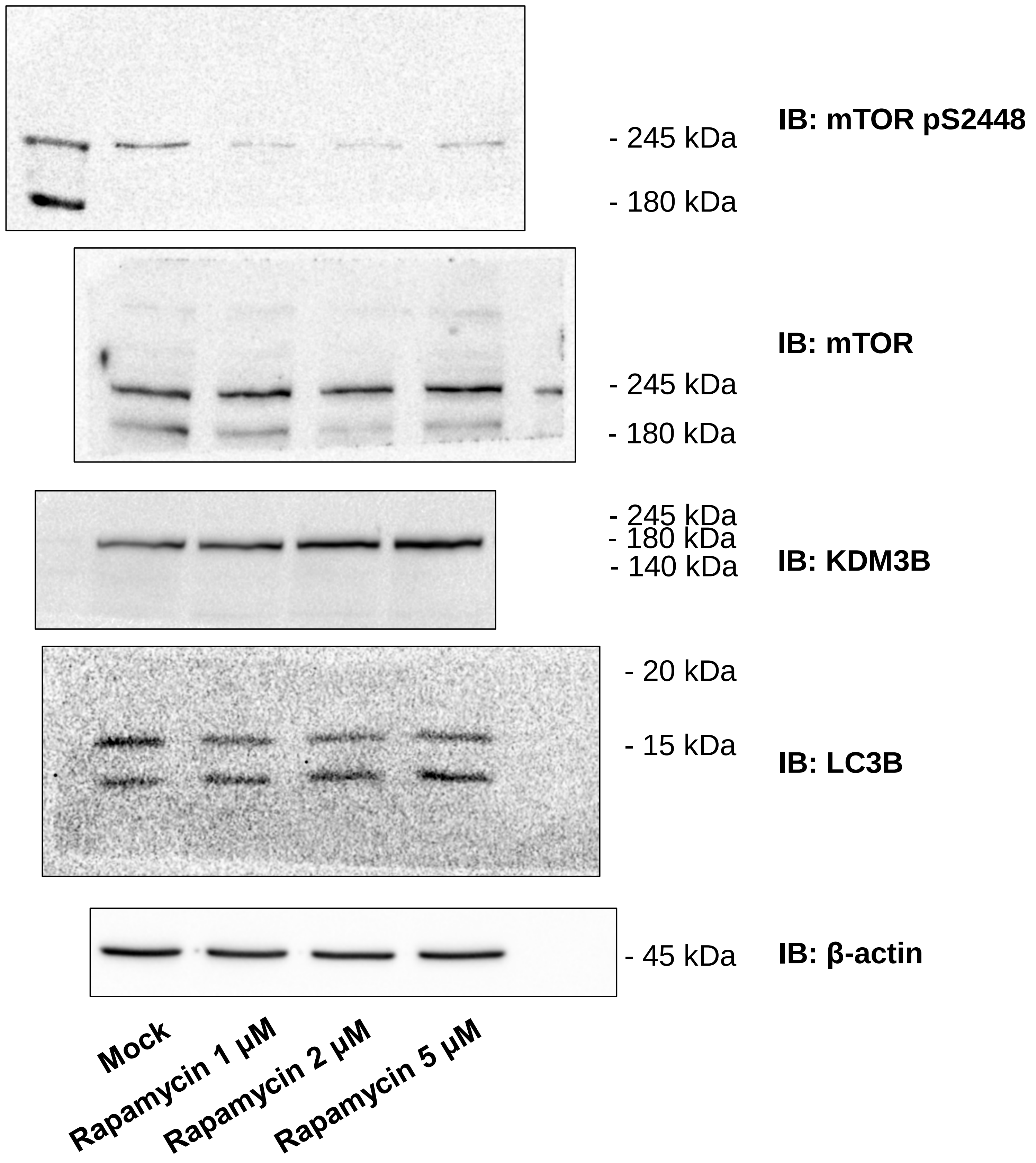

# Figure 4

**A** **The LC3B conversion in starved sh*KDM3B* cells – Western blot analysis**

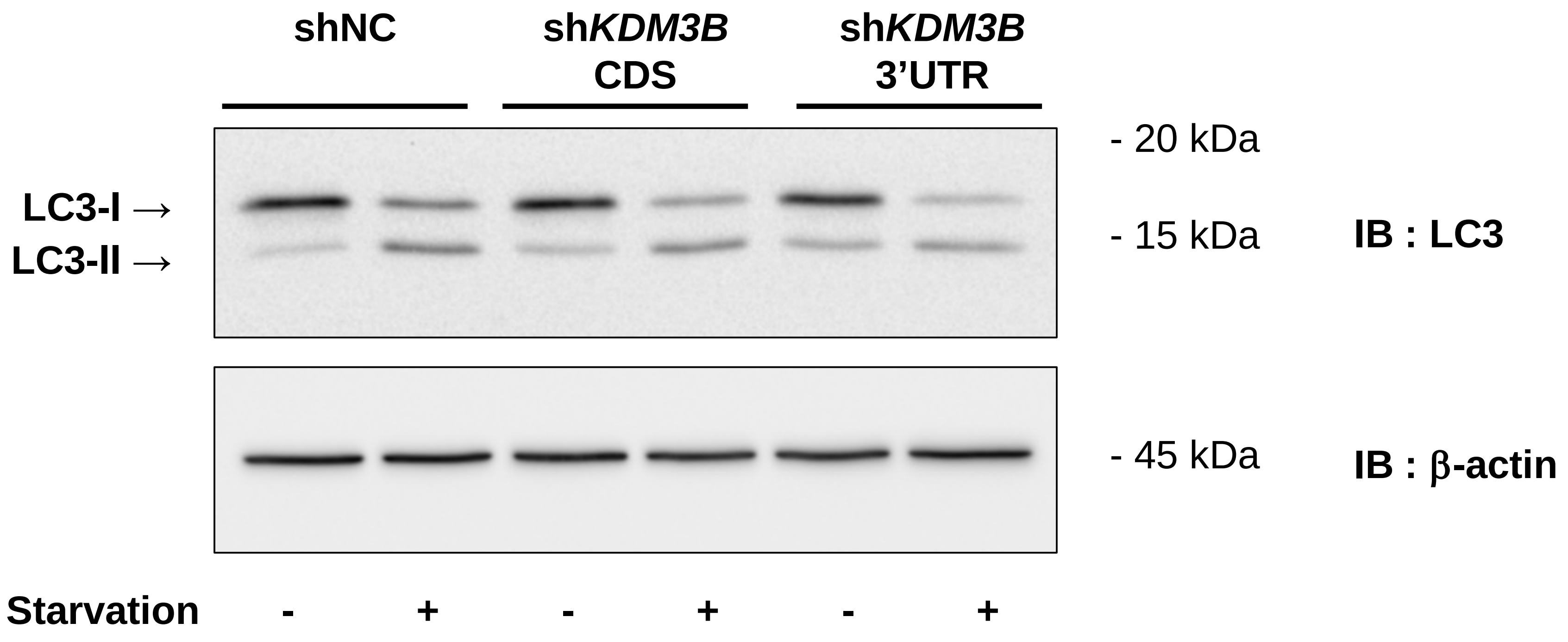


**B** **The LC3B conversion in rapamycin and/or CQ-treated sh*KDM3B* cells – Western blot analysis**

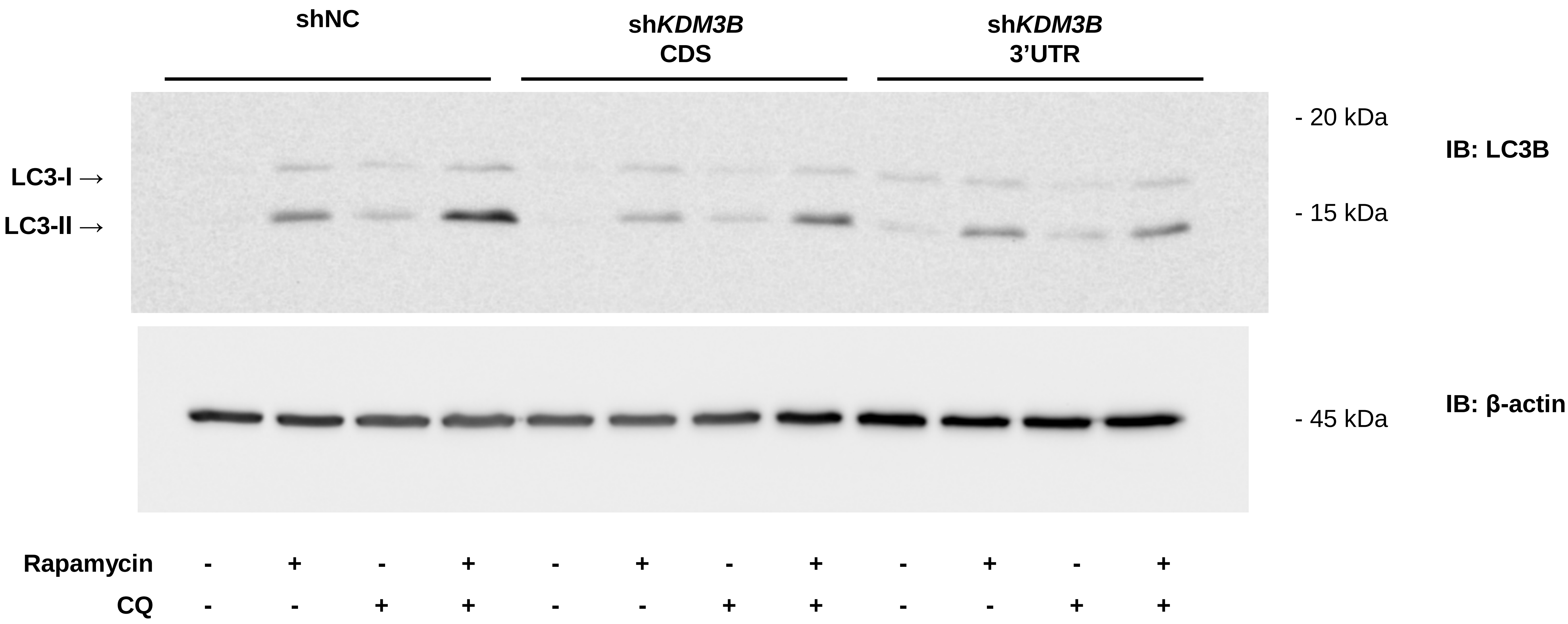

# Figure 5

**B** **The induction of KDM3B by VCP depletion – Western blot analysis**

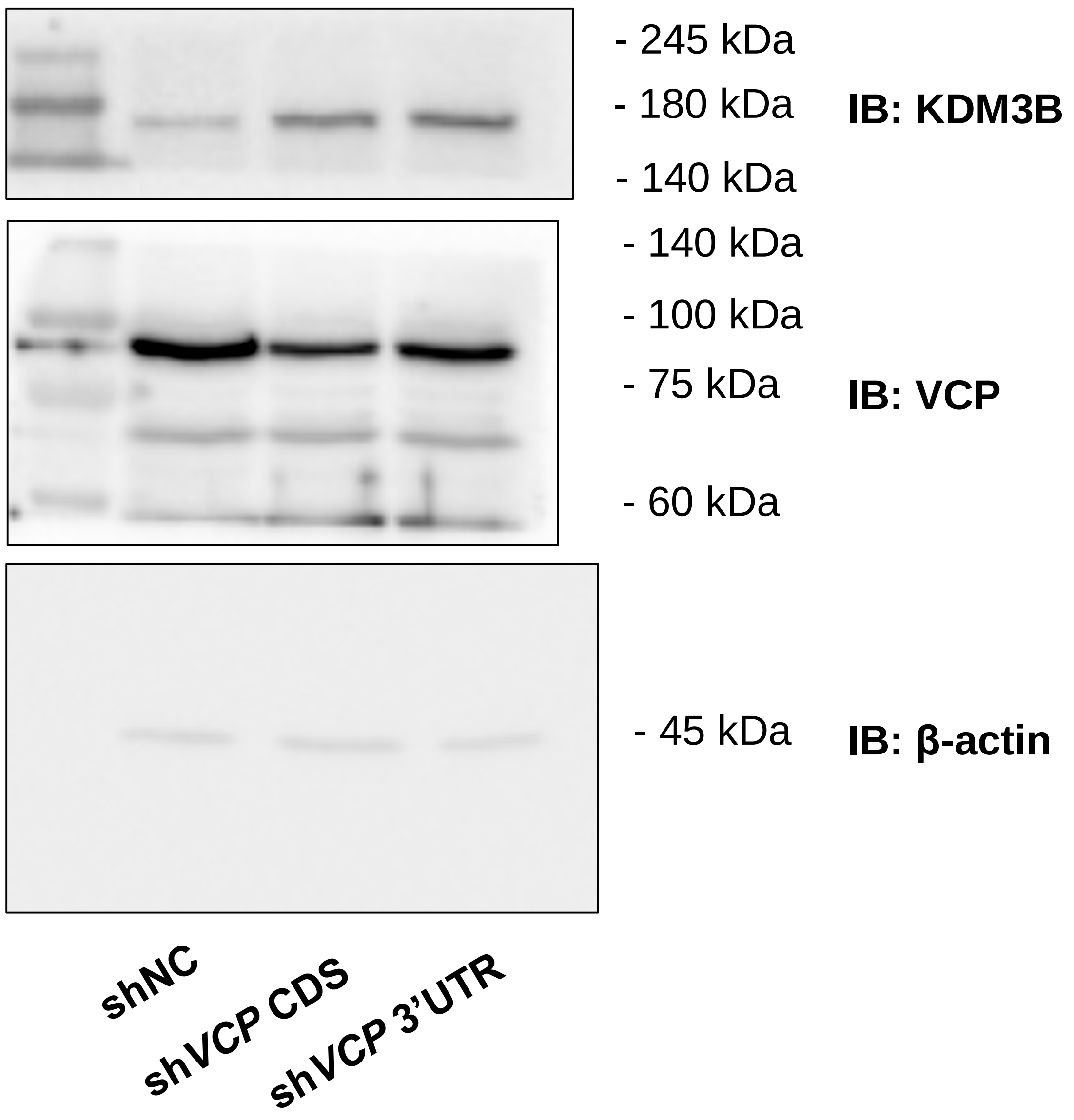


**C** **Proteasomal degradation of KDM3B by VCP – Western blot analysis**

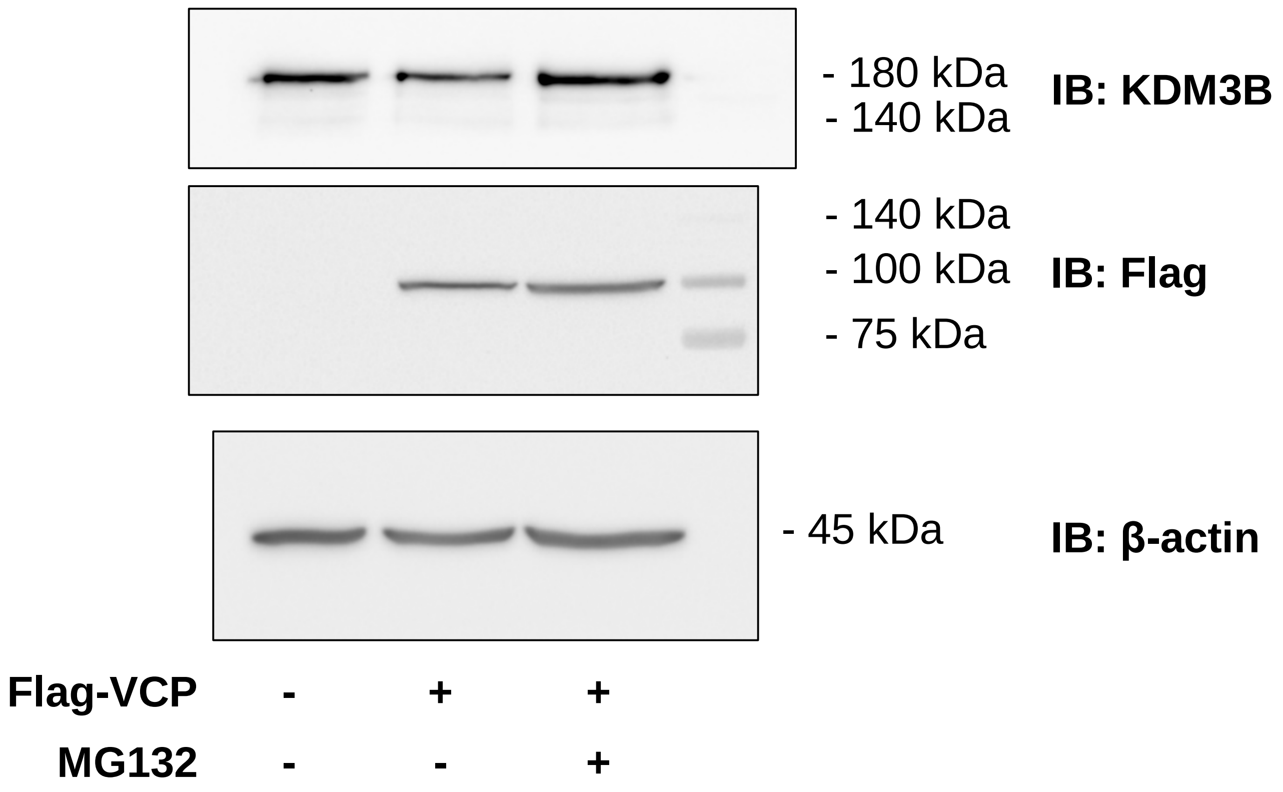


**E** **The LC3B conversion in rapamycin and/or CQ-treated Flag-VCP overexpressing cells – Western blot analysis**

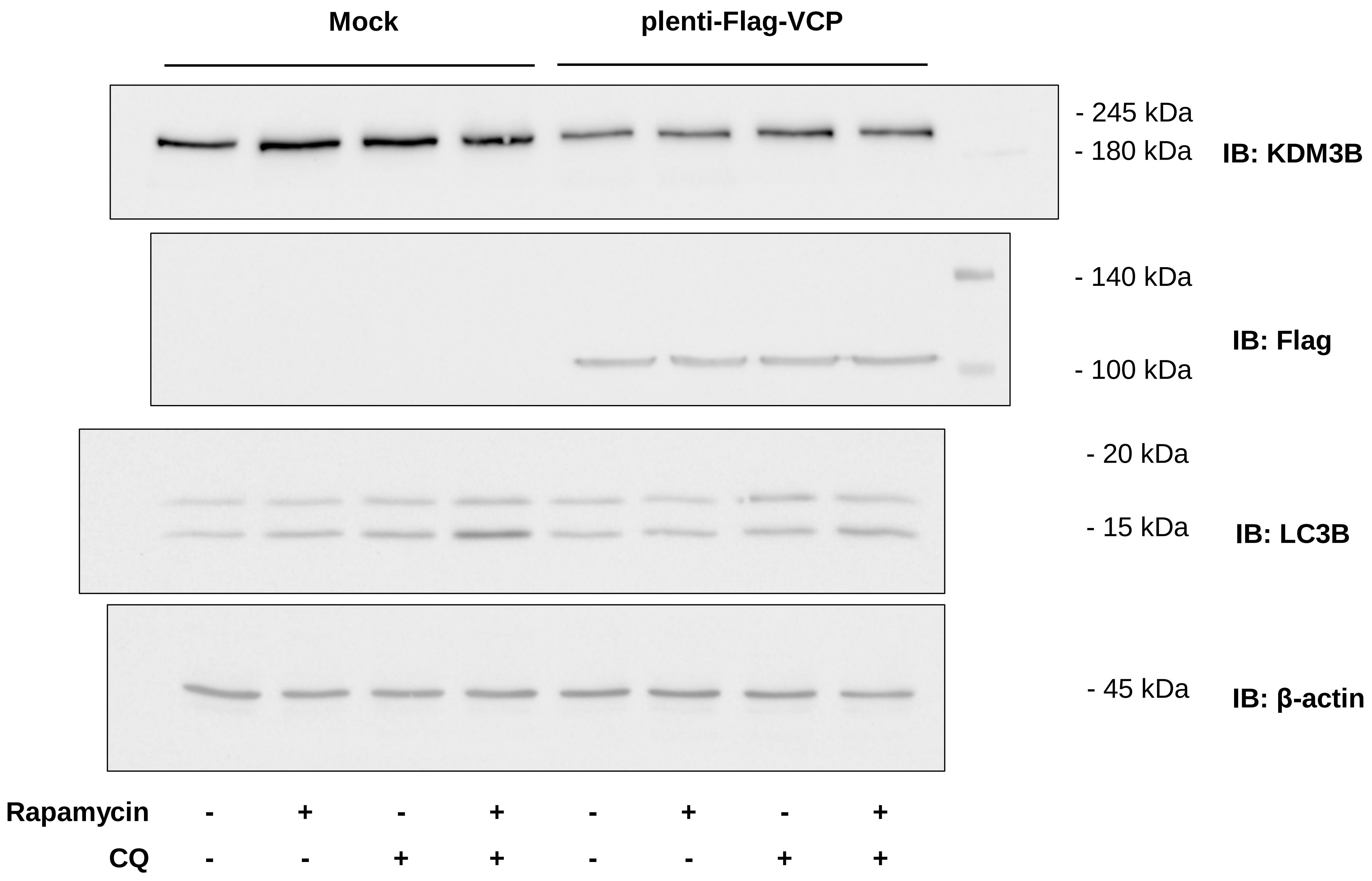

# Figure 5

## F The expression of VCP during starvation or rapamycin treatment – Western blot analysis

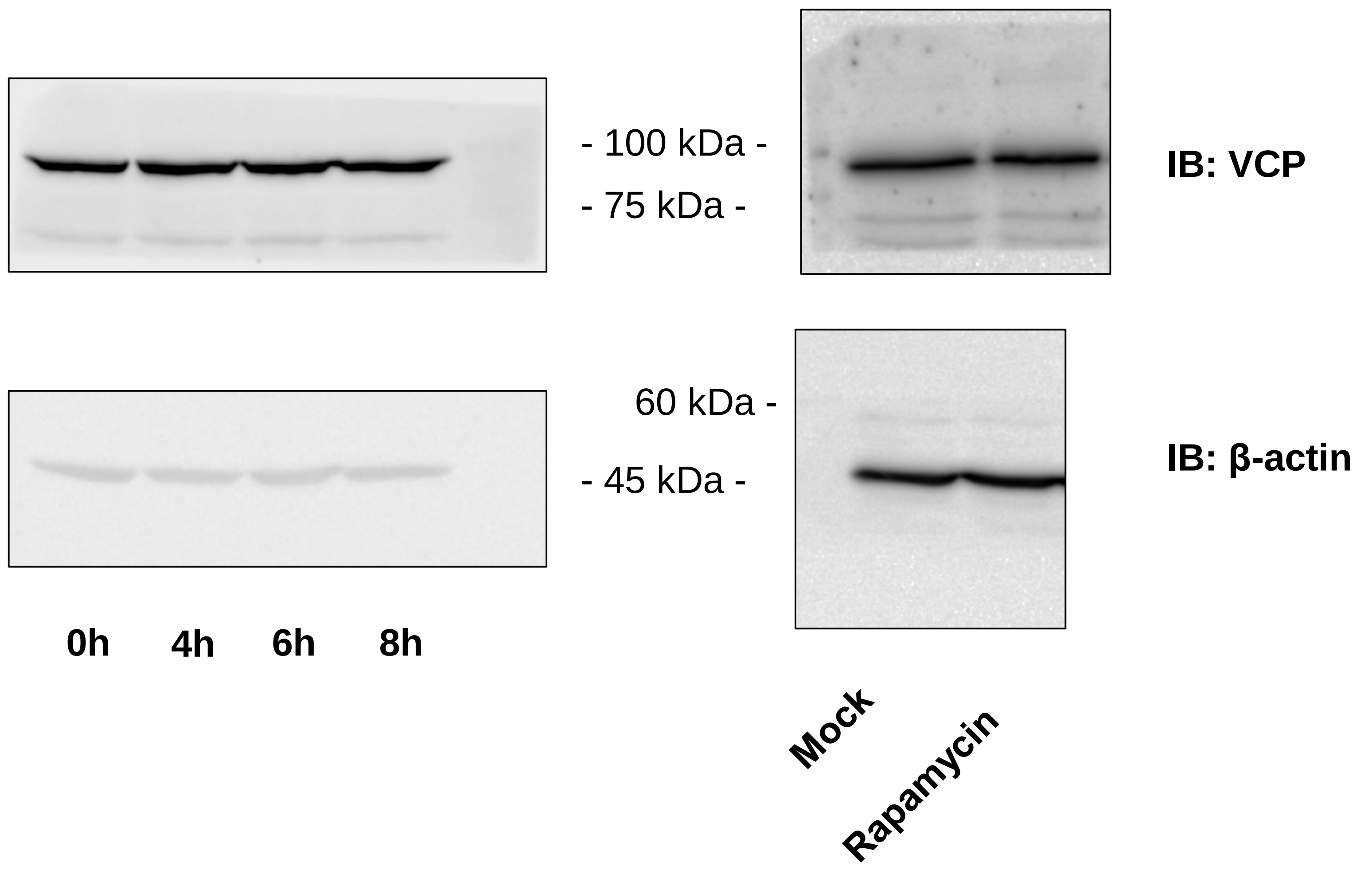


## G Interaction between KDM3B and VCP in rapamycin treated cells – Immunoprecipitation

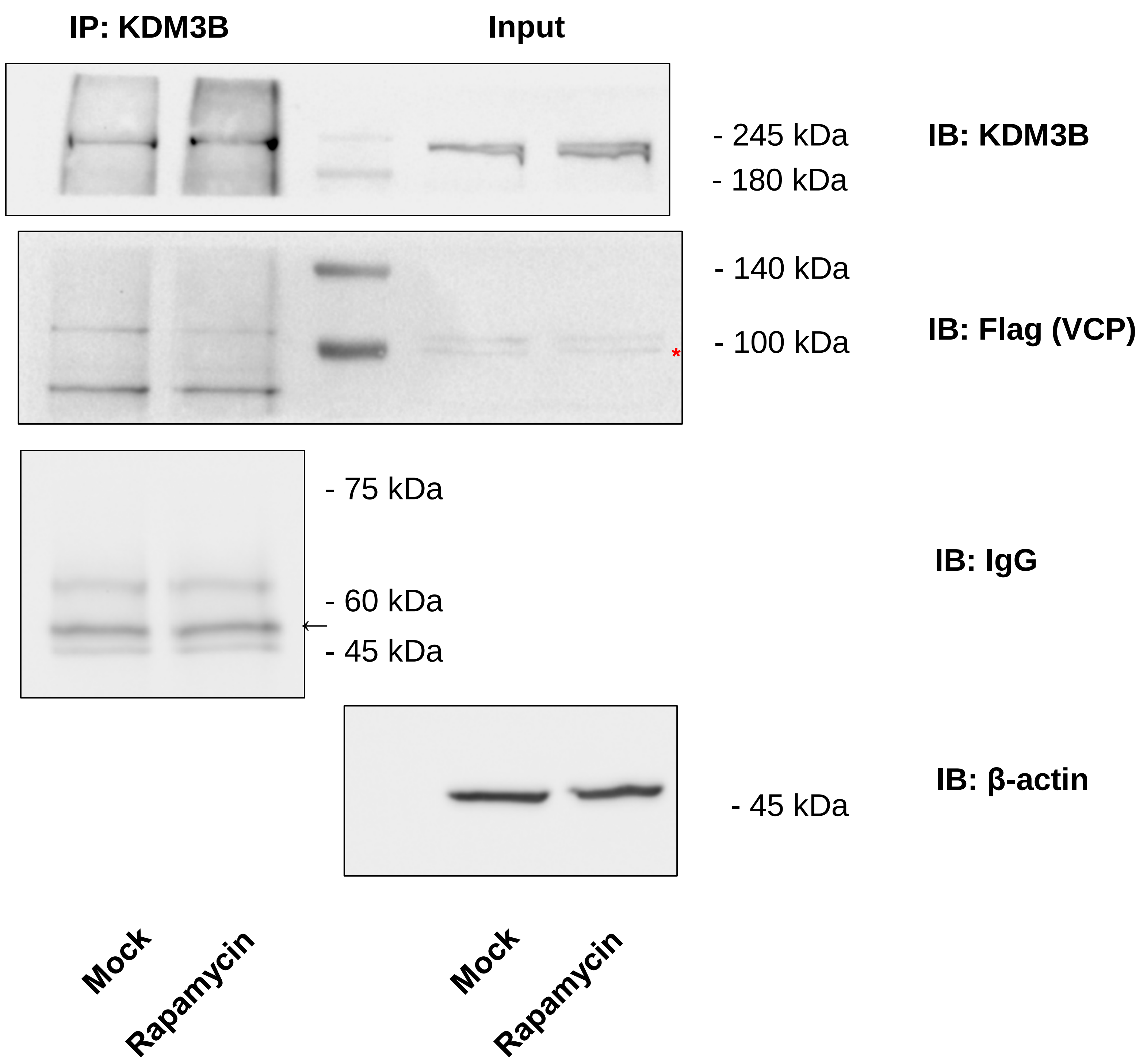

# S1 Figure

**A** The induction of ATG5 during starvation – Western blot analysis

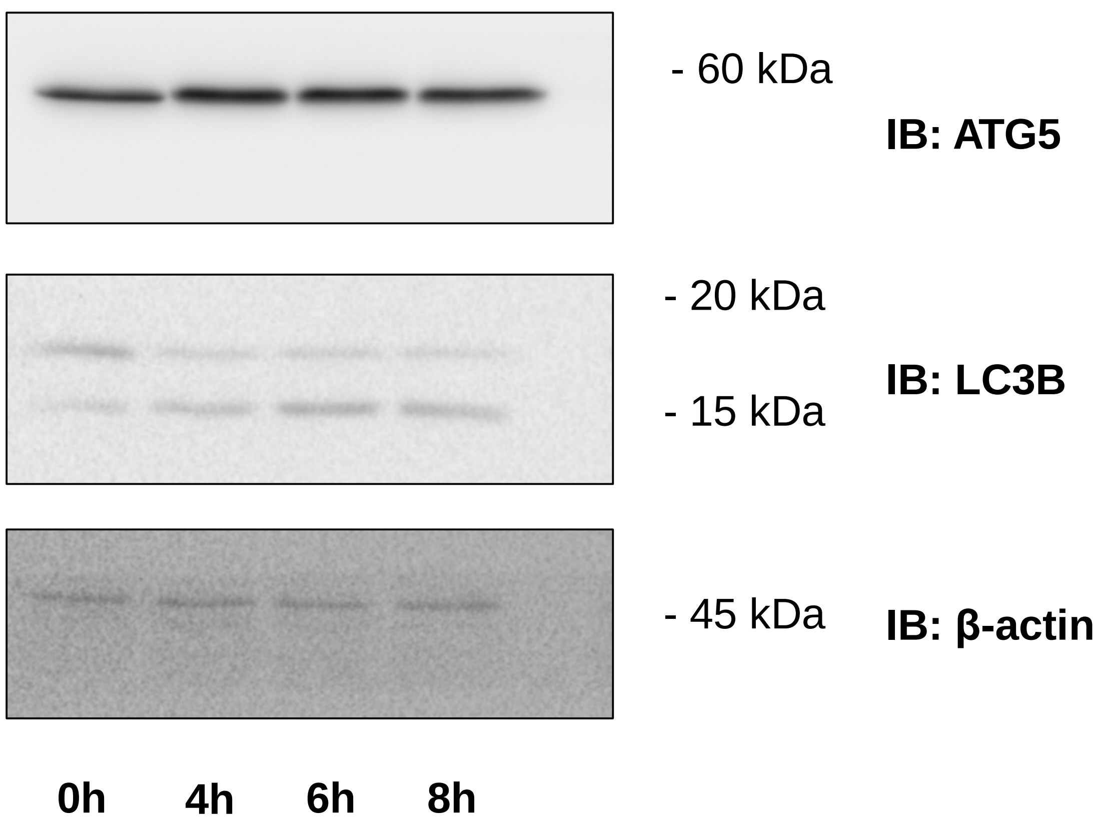


**B** Decrease of ATG5 expression by KDM3B depletion – Western blot analysis

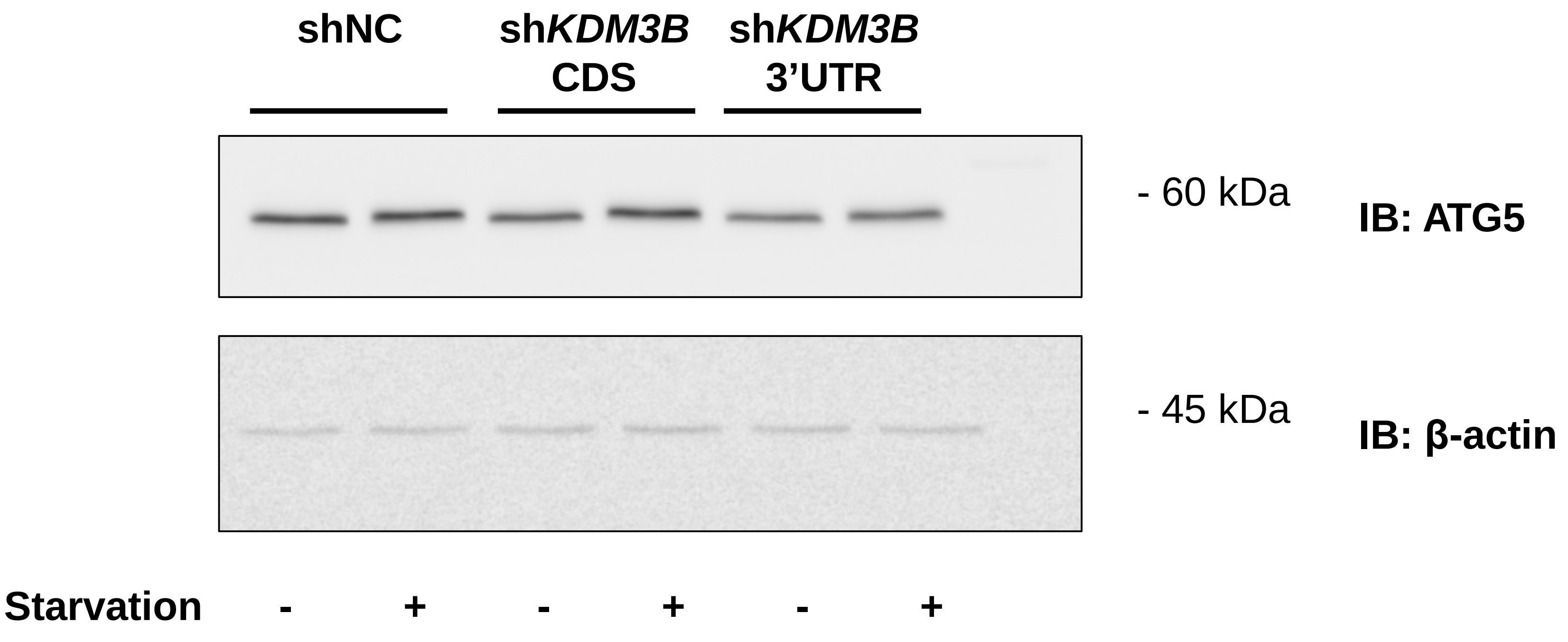

# S2 Figure

**A** **The interaction between KDM3B and VCP – Immunoprecipitation, IP : Flag-VCP**

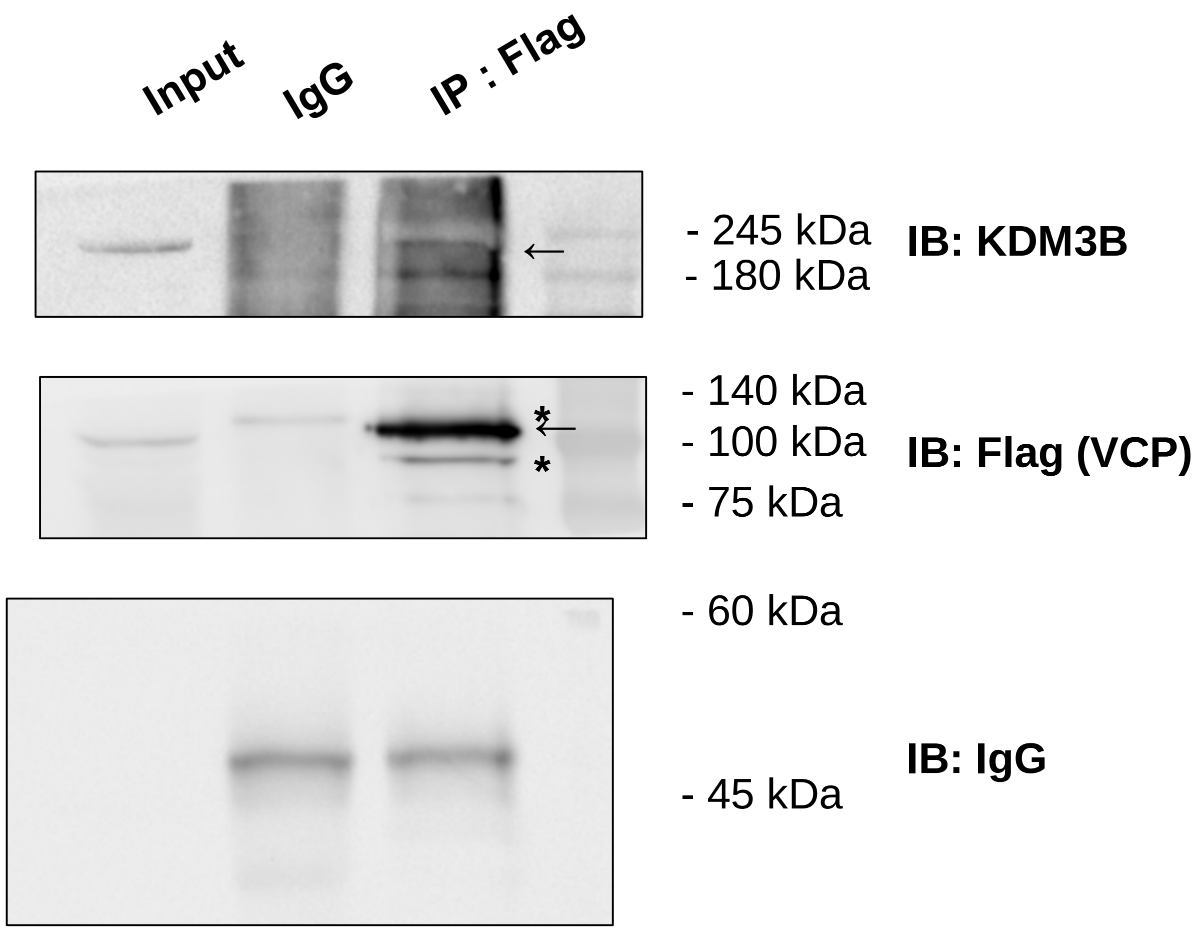


**B** **The interaction between KDM3B and VCP – Immunoprecipitation, IP :KDM3B**

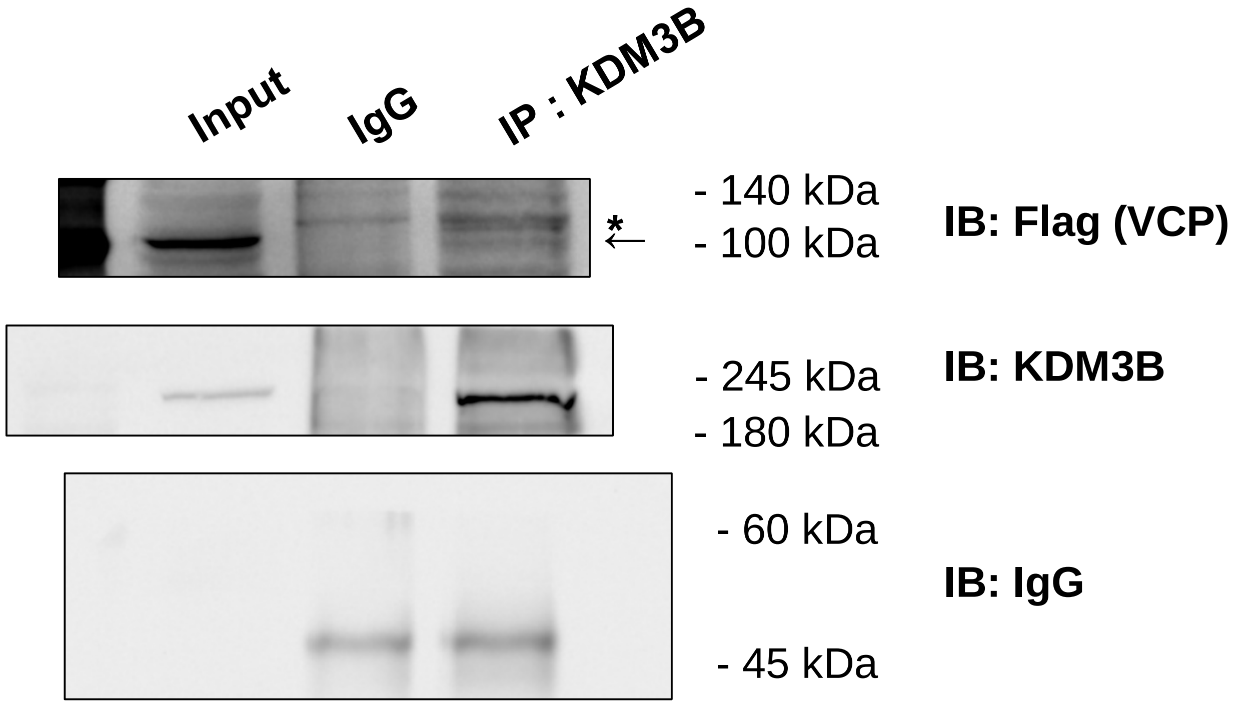

Supplement: S4 Fig — (PDF) [file pone.0236403.s004.pdf]
